# Improvement in the Usability of Meat Inspection Findings for Swine Herd Health Management

**DOI:** 10.3390/ani15050688

**Published:** 2025-02-26

**Authors:** Darko Maric, Sebastian Vetter-Lang, Johannes Klinger, Nikolaus Böhm, Karin Schwaiger, Annemarie Käsbohrer

**Affiliations:** 1 Centre for Food Science and Veterinary Public Health, University of Veterinary Medicine, 1210 Vienna, Austria; darkomaricvetmed@gmail.com (D.M.); sebastian.vetter@vetmeduni.ac.at (S.V.-L.); karin.schwaiger@vetmeduni.ac.at (K.S.); 2 Veterinary Consultant, Austria; nikolaus.boehm@aon.at; 3 Department Biological Safety, German Federal Institute for Risk Assessment, 10589 Berlin, Germany

**Keywords:** slaughter, post-mortem inspection, pig production, feedback system

## Abstract

Data from post-mortem inspections conducted using official controls on the meat production of slaughtered pigs are generally considered valuable for identifying herd health issues and ensuring meat safety. However, several studies highlighted that a multi-stage assessment of lung changes would provide more useful information on animal health than the implemented binary (yes/no) recording. For this purpose, a new scheme was developed and subsequently used by trained official veterinarians at four slaughterhouses in Austria. Implementation of the multi-stage assessment was carried out in parallel with the conventional assessment, and data collected from both schemes were analyzed and compared to evaluate effectiveness. The analysis of the data (n = 20,345) showed that the most common alteration was low-grade (28.4%), followed by moderate-grade (11.3%,) and then high-grade pneumonia (5.2%). In the case of pleurisy, 88.9% of the carcasses showed no alterations of the pleura, and 11.1% had pathological changes (low-grade pleurisy = 4.7%, moderate-grade pleurisy = 2.7%, high-grade pleurisy = 3.7%). Analysis of the results showed a strong heterogeneity of the frequency of alterations between the batches reflecting various underlying animal health issues. Among the influencing factors, the origin of the pigs had the greatest influence. The project demonstrated that the new evaluation can be carried out easily with no extra time effort once staff are trained and the technological platform for reporting is adapted. The more detailed information ensures more useful feedback is provided to the farmers and supervising veterinarians, thereby ensuing animal welfare and contributing to sustainable, improved animal husbandry.

## Simple Summary

Official veterinarians inspect meat to ensure that it is fit for human consumption; however, the meat inspection reports provided to farmers are currently limited to only yes/no answers for several pathological findings. A more detailed and standardized system for data collection is needed to increase the usefulness of the information for the primary sector. In this work, a multi-level recording system for alterations of the lungs and the pleura (no changes, low-grade, moderate-grade, and high-grade alterations) was developed. Trained staff applied this system to 20,345 carcasses slaughtered at four different abattoirs located in two different federal states of Austria. Data for 408 batches from 318 different farms were recorded in total. The analysis of the data obtained showed that frequency of pulmonary and pleural alterations was quite heterogeneous between the slaughter batches. It showed that more detailed information could be easily collected within the obligatory evaluation process. Once this standardized system is introduced on a routine basis, it can provide useful and informative feedback for farmers and veterinarians. Ultimately, the results showed that the information collected provided added value for pig farmers and the supervising veterinarians ensuring animal welfare and contributing to improved, sustainable animal husbandry.

## 1. Introduction

The average consumption of pork per capita worldwide is 34 kg per year, and both the production and consumption of pork have been constantly rising in recent years [1,2]. Austria’s mean value is slightly below this, at 33.5 kg per capita [3]. Considering these numbers, livestock production, as well as an effective and standardized meat control process, is crucial in granting security and safety to food of animal origin. Meat inspection was introduced in the mid-1880s and initially focused on detecting zoonotic diseases such as trichinellosis and taeniasis [4]. Over the years, meat inspection processes have been expanded and refined. For example, post-mortem meat assessment shifted mainly from manual to visual inspection of carcasses to prevent cross-contaminations [5], and regulations have been enacted to ensure the provision of food chain information (FCI), which aims to guarantee the safety, transparency, and traceability of food intended for human consumption [6]. In the European Union (EU), the requirement for a database was established in 2004 by Regulation (EC) 2004/854 [7], which requires, among others, that the results of post-mortem inspections and tests are to be included in relevant databases.

Prior to 2008, in Austria, findings in the context of meat inspection were recorded by hand, which made it difficult to evaluate and compare the information. This problem was solved by the introduction of electronic recording in 2008. In Austria, the data are now electronically recorded and uploaded to the Austrian Meat Inspection (Österreichische Fleischkontrolle—ÖFK) server. From there, the information is forwarded to the Consumer Health Information System (Verbrauchergesundheitsinformationssystem—VIS) with the information stored in its database [8]. Moreover, the EU regulations for conducting official controls and recording the outcome of the inspections were updated in 2017 and 2019 [9,10].

The examination of slaughtered animals and post-mortem meat inspection not only serve to control food hygiene and monitor animal welfare but also provide an important data source for determining the health status of animals. Analysis of the data from 2016 showed that the most frequently recorded pathological alterations affected the respiratory tract, with pneumoniae comprising the majority of findings [11]. The porcine respiratory disease complex is characterized by the interaction of various primary infectious agents, such as the Reproductive and Respiratory Syndrome virus, Influenza A Virus, *Mycoplasma (M.) hyopneumoniae*, and *Actinobacillus (A.) pleuropneumoniae*, which may lead to opportunistic infectious agents [12,13]. Merialdi et al. (2012) reported that enzootic pneumonia (EP)-like lesions, characterized by cranioventral pulmonary consolidation and caused by many different bacteria, particularly *M. hyopneumoniae* and *Pasteurella (P.) multocida*, and pleuritis are the most frequently found in pig lungs at slaughter [14]. Pleural lesions are commonly detected at the abattoir due to the chronic nature of the pleuritis, which can take 3 months or more [15]. The most important cause of chronic pleuritis in pigs is *A. pleuropneumoniae*, but several other bacterial pathogens may also be involved, particularly *Haemophilus parasuis*, *P. multocida* and *Streptococcus suis* [16,17]. Cranioventral pleural lesions are strongly associated with complicated EP-like lesions, whilst dorsocaudal lesions are considered suggestive of recovered pleuropneumoniae, severely affecting respiratory function [14].

To allow detailed documentation of alterations of the respiratory tract at slaughter, specific evaluation schemes have been studied to provide a comprehensive picture of a farm’s respiratory health status. For example, Goodwin et al. 1969 [18] developed a lung lesion evaluation scheme focused on lesions typically caused by enzootic pneumonia. Pessoa et al. 2021 [19] combined data collection at the farm level with individual scoring of lungs for pneumonia, scarring and dorsocaudal and cranial pleurisy lesions at slaughter, and confirmed that both coughing and lung lesions were largely pen-specific, which fit the disease presentation of *M. hyopneumoniae*. In contrast, coughing in weaners was not reflected in a higher prevalence of lung lesions at slaughter.

As pulmonary infections and pleurisy have a healing phase of several weeks to months depending on the causative origin [20], there might serious impact on the carcass weight and the meat quality, which lead to significant economic losses during the fattening period. For example, a study from 2015 analyzed the impact of 18 post-mortem findings on the average daily weight gain in pigs for fattening. The results showed that the expected average daily weight gain decreases significantly for pigs with at least one of the post-mortem findings: 13 g daily for severe pneumonia and 7 g for visceral pleuritis [21].

For this reason, a broader assessment of pulmonary changes and feedback to the animal keeper would be sensible. During meat inspection, in Austria the assessment of pulmonary abnormalities in slaughter animals currently concentrates on a binary decision (yes/no), with a main focus on suitability for food production. Several schemes have already been published in order to record pulmonary lesions and provide information meaningful for farm health assessment. For example, in the lung lesion scheme of Madec and Kobisch [22] published in 1982, each lobe is divided into four quarters and the number of affected quarters of all lobes is summarized. Another more sophisticated data-based scheme was published by Christensen et al. [23] in 1999, where lung lesions were evaluated depending on the weight of the altered lung lobe and the results reported on a 100-point scale. In contrast, the score published by Blaha et al. [24] in 1994 assessed the proportion of the total visible lung area which is affected by pathological/anatomical lung lesions. On the basis of this, alterations due to pneumonia were scored low (surface-related extent of lesion ≤ 10%), moderate (surface-related extent of lesion 11–30%), or high (surface related extent of lesion > 30%). Steinmann et al. (2014) [25] measured the surfaces of macroscopically unaltered lungs of pigs with an average slaughter weight of 100 to 110 kg and calculated for each lobe the percentage of the total lung surface. Usage of this simplified lung scheme demonstrated the benefits of using a standardized base for evaluating lung lesions and training the meat inspectors. In addition to the method of measuring the magnitude of the alterations, among the described schemes, completely healthy lungs are handled differently. Sometimes, lungs without lesions (without any observable findings) are recorded separately, whereas in other schemes they are merged with lungs having lesions extending to less than 10% of the total surface area [25].

In addition, various studies showed that the current evaluation method of lung lesions is not sufficiently reliable, as significant inter-rater disagreement and unexplainable variation between different abattoirs were observed [26,27]. Furthermore, as recorded patterns of pathological findings differed within slaughterhouses, further harmonization of the recording was considered essential to provide high-quality feedback to farmers and veterinarians [11].

In practice, the current situation results in a lost opportunity to collect additional valuable information, and the data provided ultimately lack sufficient significance. A multi-stage assessment system combined with feedback to farmers could lay the foundation for improved consultation with the attending veterinarian. This could subsequently have a direct influence on corrections in farm management.

To facilitate improved herd health assessment, the objective of this study was to increase the reliability of official meat inspection data by using a detailed and standardized scheme to evaluate surface-related lung and pleural lesions of slaughter pigs during routine meat inspection in conventional slaughterhouses in Austria. Based on experiences related to the practical application of the more detailed scheme and evaluation of the reliability of the recorded data, a recommendation should be developed regarding data use and implementation for swine herd health management by farmers and veterinarians.

## 2. Materials and Methods

### 2.1. Description of the Evaluation Scheme

In the first phase, attention was given to the description of a new scheme suitable for routine application which was based on the work published by Steinmann et al. in 2014. This included finetuning of the definition of individual categories and subsequent implementation through discussion and training sessions with veterinarians involved in meat inspection. Given that the lungs exhibit some of the most common pathological changes during post-mortem examinations [11,28,29], the focus of the new scheme was on evaluation of pathological alterations of the lung and the pleura using the codes for pneumonia and pleurisy. Whether the pigs were raised under organic/free-range conditions or conventional, indoor conditions was not considered [30]. To collect more detailed information on lung alterations, the findings ‘pneumonia’ and ‘pleurisy’ were categorized into three severity grades each based on the surface area affected by the pathological change as suggested by Steinmann et al. (2014) [25]. The percentage of the total lung surface was estimated for minor and main lobes as a reference base for the suggested classification system. Furthermore, it was clarified that atelectasis was not recorded within the system, and alternative available codes used for artifacts of the lungs affected through technical issues (e.g., through scalding water, blood aspiration). Regarding the pleura, preferential assessment of the carcass halves was recommended; however, it was also accepted that it would be assessed on the organ side as only one person had to examine both the lung and the pleura. Because the examinations at slaughterhouses have to be conducted under time constraints, no further detailed recordings of the various forms were expected. To ensure that the classification of the different grades was standardized and independent of the examiner, reference values for the three stages were agreed on. The division of severity grades was implemented for pneumonia as follows: no alteration, low-grade (>0–10%, reference value: one minor lobe of the lung is approximately 10%), moderate-grade (>10–30%, reference value: one main lobe of the lung is approximately 25%), and high-grade (>30%). Similarly, for pleurisy, no alteration, low-grade (>0–10%, reference value: one palm is approximately 10%), moderate-grade (>10–30%, reference value: one plate is approximately 25%), and high-grade (>30%) were defined.

During training, the official veterinarians evaluated pictures and videos, and then a questionnaire was filled out to assess the agreement of their evaluations. The results of this exercise were reviewed at a project meeting, where differences in the results evaluation were discussed. This served to balance out identified uncertainties in the assessment so that the scheme was implemented uniformly in the project. In a final meeting with the involved meat inspectors, their perception of the practicability was assessed.

The new scheme was compared to the current Austrian system that is laid down in national legislation (Meat Inspection Regulation) which requires that a record is made using predetermined codes for partial alterations of the lung or pleura, e.g., pneumonia (E167), pleuritis (E169), or foreign body in the lung (E191).

### 2.2. Data Collection

Four slaughterhouses, two in Upper Austria and two in Lower Austria, were recruited to participate in the project (marked as SH01, SH02, SH03, and SH04) and the relevant agreement was collected. For each of the slaughterhouses, one coordinating vet was identified through the supervising regional ministry and, among the respective staff, several meat inspectors were recruited to specifically perform the assessment of the carcasses according to the new protocol. The trained veterinarians applied the new scheme on various days independently and in parallel with colleagues applying the established recording system.

Due to practical reasons, for each assessor, the time period was fixed on a day when the project-specific recording was running, with all the pigs slaughtered in that time period being included. Covering only slaughter batches of a minimum size from predefined farms would have required the permanent availability of additional staff, as the exact slaughter time is not fixed beforehand and the decision to deliver pigs to slaughter is also made on short notice.

Within a few months (November 2022 to January 2023), the new scheme was applied to 20,345 carcasses. A total of 408 batches from 318 different farms were assessed using the new scheme. The number of animals per batch included in this study varied from single animals to a maximum of 361 animals. The average size of the investigated batches was around 50 pigs. The evaluations were carried out by 8 veterinarians in a total of 61 sessions. On average, each session lasted 3.5 h, with 333 carcasses assessed and the ID of the farm of origin for allocation to batches collected. Average slaughter capacity of the involved slaughterhouses was around 100 to 200 pigs/hour. Additional data on the farms were not available. Because the current (also named old) and new schemes were applied at the same time but by different persons, in principle the data of the different assessments could directly be compared. In order to follow data protection requirements, a declaration of consent had to be obtained from the respective pig suppliers for using the data collected according to the old scheme. A total of 46 farmers signed the declaration of consent, and data for a subset of 5268 carcasses classified using both schemes were available for comparative investigation. As these 46 farmers agreed to use data for all pigs slaughtered in a specified time frame, 17,589 findings from other batches were released from the same farms according to the old scheme. Thus, a total of 37,934 findings were available, which can be divided into three groups: carcasses that were only assessed according to the new scheme (n = 15,077), carcasses that were assessed according to the new and old schemes (n= 5268), and carcasses that were only assessed according to the old scheme (n = 17,589).

### 2.3. Data Analysis

After the data were digitally recorded, they were analyzed with the R software (version 4.4.1 [31]). First, basic methods of descriptive statistics for pneumonia and pleurisy were applied: the minimum, mean, median, maximum, and quantiles (Q1 and Q3) were calculated for the different data sets as described above.

Batches with fewer than 50 carcasses were excluded in detailed analyses on the batch level. For this purpose, a batch was defined as a group of pigs delivered from an individual pig producer to the same slaughterhouse on the same day. For comparison of different subsets, the Spearman correlation test was applied as specified with results.

To analyze influencing factors on the pathologies of pleuritis and pneumonia in the new scheme, rated with three severity levels, multinomial models (R-package nnet [32]) were calculated and included the fixed effects of slaughter plant, rater, farm, and batch to assess the effect those variables had on the assessment of the carcass. The effect was quantified as the %-reduction in the proportion of variance explained by the model (R^2^) in models that excluded one of the four variables compared with the full model, including all four fixed effects. For this analysis, only batches ≥50 animals were used, resulting in a data set of 15,156 carcasses that were assessed based on the new schemes (in 170 batches from 128 farms that were slaughtered in four slaughter plants and assessed by eight veterinarians).

To assess the probability of a positive finding when evaluated according to the old scheme depending on the finding based on the new scheme, mixed binomial models (R-package lme4 [33]) were calculated, including the nested random intercepts of the slaughter plant/rater and farm/batch, in addition to the fixed effect of the classification based on the new scheme. For pairwise comparison between different severity levels of a positive finding based on the new scheme, a post hoc test was performed (R-package multcomp [34]). For this analysis, data were used from 5268 carcasses that were assessed based on both schemes (in 66 batches from 46 farms that were slaughtered in two slaughter plants and assessed by three veterinarians).

## 3. Results

### 3.1. Application of the New Scheme

**Figure 1:**
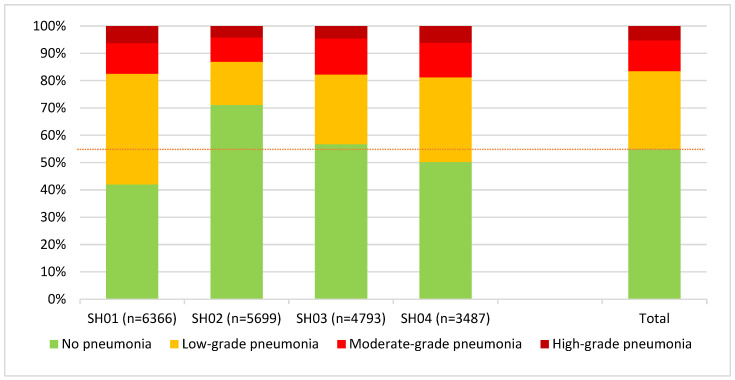
Distribution (proportion of all carcasses) of pneumonia findings according to the new scheme, also broken down by slaughterhouses (numbered SH01 to SH04). The average prevalence for carcasses with no pneumonia (55.1%) is displayed as a dotted line.

Evaluation of the entire data pool of 20,345 carcasses assessed according to the new scheme showed that 55.1% of the animals had no alterations of the lung. However, in most of the animals with pneumonia, low-grade forms were observed in 28.4%, followed by moderate-grade forms in 11.3%, and the rarest was high-grade pneumonia in 5.2%. Analysis of the data individually for the four different abattoirs showed specific patterns with deviations from the average, particularly in the “No pneumonia” category, where the difference between SH01 and SH02 was 29.1% (42 versus 71.1%), as shown in Figure 1.

**Figure 2:**
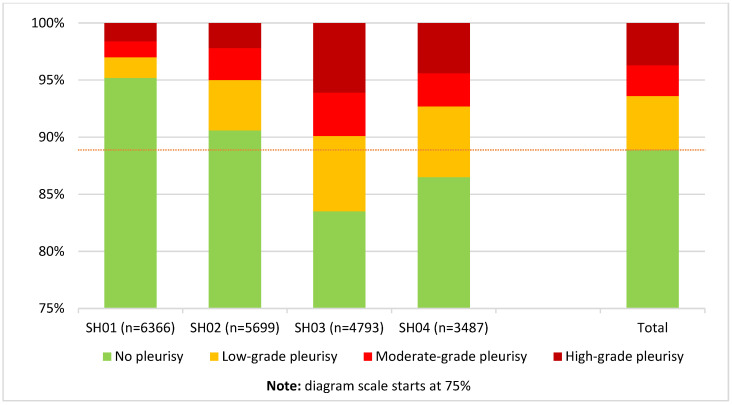
Distribution (proportion of all carcasses) of pleurisy findings according to the new scheme, also broken down by slaughterhouses (numbered SH01 to SH04). The average prevalence for carcasses with no pleurisy (88.9%) is displayed as a dotted line.

In the case of pleurisy, the analysis of the data set showed that 88.9% of the carcasses examined showed no alterations of the pleura. Here, as with pneumonia, the most frequent changes were low-grade in 4.7%, but in contrast, high-grade pleuritis in 3.7% was more common than the moderate-grade pathology in 2.7%. For the various abattoirs, there were some deviations from the overall value, as shown in Figure 2.

When assessing the link between the recordings of pneumonia and pleurisy, cross-tabulation showed (Table 1) that the combination with the same severity grade was always the most frequent. However, the analysis also shows that the severity of one of the alterations might be quite different from the other one. For example, high-grade pneumonia was recorded for 38% of the animals with no recorded alteration of the pleura. On the other hand, high-grade pleurisy was observed for 3.5% of the animals without any alterations of the lung.

The Spearman correlation test applied to the pathological alterations by severity grades showed a weak but significant positive correlation (r = 0.18, *p*-value < 0.005) between pneumonia and pleurisy. The binary correlation of those pathologies (i.e., only considering positive finding yes/no, irrespective of severity) yielded the same result (r = 0.13, *p* < 0.001).

Analysis of the impact of influencing factors (Table 2) showed that the individual meat inspector only has a marginal influence on the occurrence of both pathologies; the slaughterhouse has virtually none. The farm has the greatest influence, although this remains limited for both pleurisy and pneumonia. It explains almost 14.3% and 18.5% of the variance in the occurrence of pneumonia and pleurisy, respectively.

### 3.2. New and Old Schemes in Comparison

**Figure 3:**
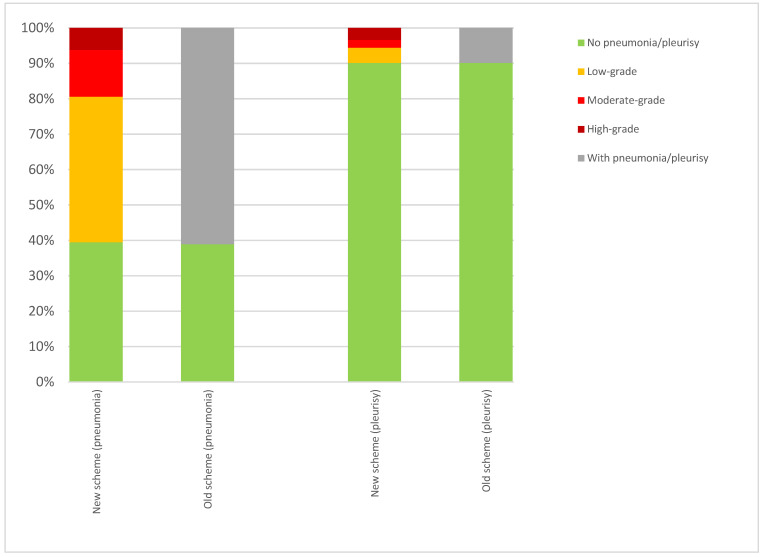
Percentage of alterations for pneumonia and pleurisy with new and old schemes. Only those animals where data for both schemes are available are included (n = 5268).

Among those animals assessed with both schemes, the percentage of animals affected according to the new and old schemes was similar for the recording of pneumonia (60.5 and 61.1%) and identical for pleurisy (9.9% each) (Figure 3).

**Figure 4:**
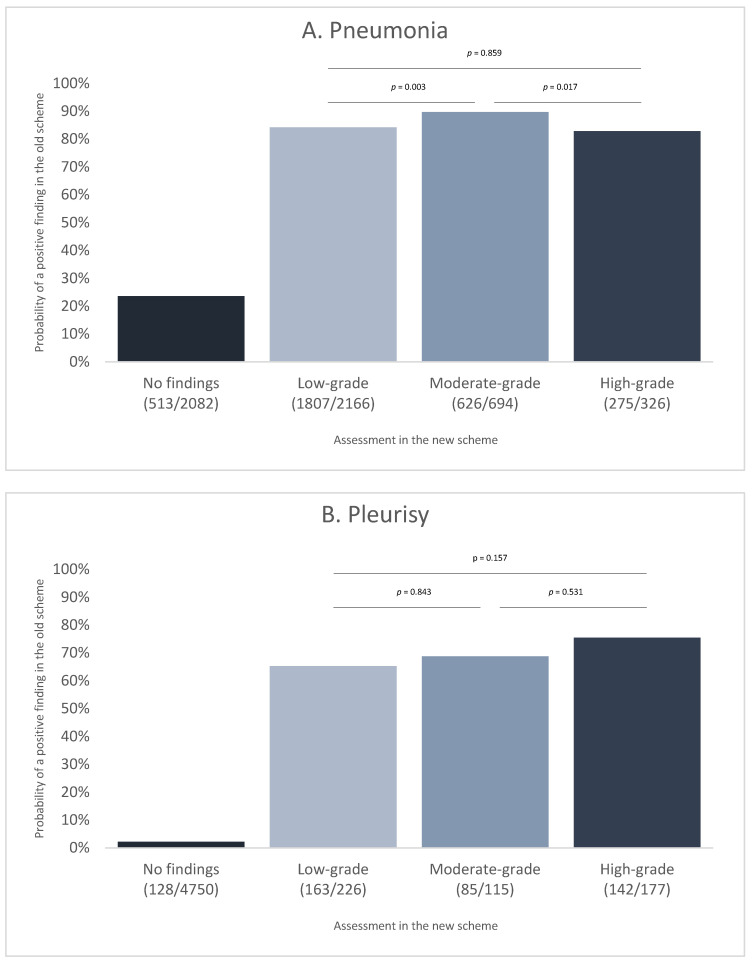
Probability of a positive finding in the old scheme for the different groups as a result of categorization according to the new scheme. (**A**) Data for pneumonia. (**B**) Data for pleurisy (in brackets: positive carcasses for the old scheme/carcasses in the category according to the new scheme). The *p*-values shown in the figures are taken from the respective post hoc test of a model explained in Section 2.3.

To go into more detail, for each individual animal assessed with both schemes, the recordings were compared to assess the correlation between a positive finding in the new and old scheme. This analysis showed some discrepancies between the records of the severity of a pathology using the new scheme and the probability of positivity in the old scheme (Figure 4). For example, 23.6% of the animals with no record for pneumonia in the new scheme were reported positive in the old scheme (Figure 4A). On the other hand, around 80 to 90% of the animals with findings of pneumonia in the new scheme were also positive in the old scheme. For pleuritis, the difference for a positive record for those carcasses with findings in the new scheme was more pronounced, with 65 to 75% of positive animals also identified with the old scheme (Figure 4B). Statistical analysis did not show a significant trend for the severity of findings in the comparison of the two schemes, high-grade pathologies based on the new scheme were not statistically significantly more likely detected based on the old scheme compared with low-grade pathologies based on the new scheme.

**Figure 5:**
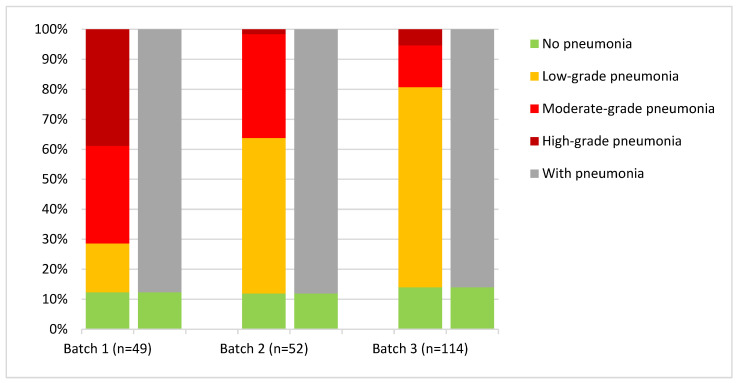
Three batches with similar percentages of any type of pneumonia findings (87.7, 88.1, and 86%). The number of animals is given in brackets. The left side is the assessment with the new scheme and the right side is the assessment with the old scheme. A clear difference in severity of findings between the batches compared is visible when the new scheme is applied to the same animals in the slaughter batch.

To reflect in more detail the benefits from additional information on the proportion of the individual grades noted with the new scheme in comparison with the old scheme, data for three exemplary batches that were tested with both schemes are displayed in Figure 5. They appear very similar at first glance when comparing the proportion of lungs with no pneumonia using both schemes; however, when the positive findings for the same batches are displayed with the new scheme, a clear difference in the distribution of the severity of the alterations displayed by the individual batches becomes visible.

### 3.3. Application of the Old Scheme

Austrian Meat Inspection (Österreichische Fleischkontrolle—ÖFK) provided data on 17,589 carcasses from the 46 farms for an extended period of time that were assessed in addition to those 5268 animals that were classified under both schemes. Overall, in this data set, 50.6% of the animals had some form of pneumonia, and 11.6% had pleurisy.

**Figure 6:**
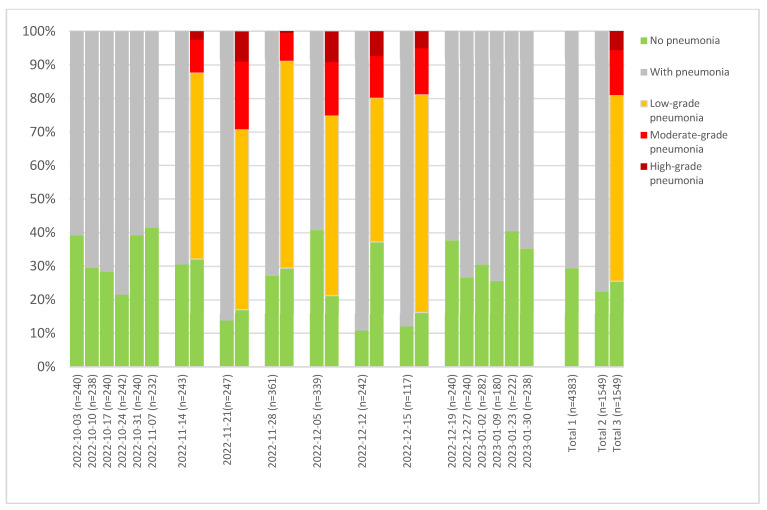
Results from farm 1, which delivered animals on 18 different days. A total of 4383 animals were involved, and six batches between 14 November 2022 and 15 December 2022 were evaluated according to both schemes. All findings were collected in SH01. Total 1 shows the average of all 18 batches according to the old scheme. Total 2 shows the average of the 6 batches according to the old scheme that were also evaluated with the new scheme. Total 3 is the average of the 6 batches according to the new scheme.

**Figure 7:**
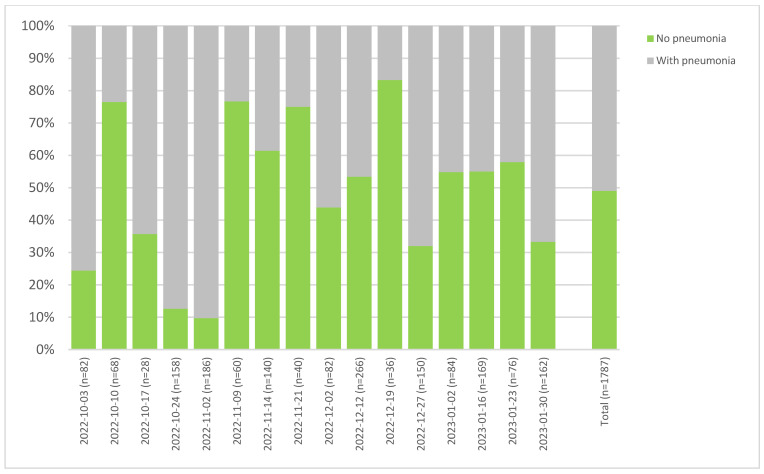
Results from farm 2, which delivered animals on 16 different days. A total of 1787 animals were involved, all of which were assessed by the old scheme. Findings were collected in SH04.

In order to obtain more detailed insight into the patterns, we analyzed two farms delivering a large number of pigs on a regular basis over the extended time period to the same slaughterhouse, each according to the old scheme, broken down by slaughter batch (Figure 6 and Figure 7). Although the batches were delivered on a weekly basis, the health status of the batches was quite heterogenous. It can also be seen that the two farms show strong differences regarding pneumonia findings, with farm 1 (Figure 6) exhibiting average findings of 70.5%, whereas the farm 2 showed 51% (Figure 7). For the latter, very large differences in the health status of the animals are sometimes visible between consecutive batches.

For six batches (Figure 6), data were available for both schemes, whereas for four batches, the percentage of ‘no findings” was similar, and for two batches, a difference in both directions was clear. Furthermore, it becomes clear that high-grade pneumonia occurs to varying degrees in these batches.

### 3.4. Assessment of the Practicability of the New Scheme

There was consensus among the meat inspectors involved, namely, that recording of pathological findings of the lung and pleura according to the new scheme can be applied without extra time efforts in the slaughterhouses involved if the recording system allows easy access to the codes. To further assist this process, it was suggested to reflect the severity grades on the screen by giving severe lesions a darker color than the less severe ones. Discussion also confirmed that more than three severity grades were not considered necessary. In contrast, a system where different types of alterations of the lung would have to be recorded was perceived as difficult to apply on a routine basis.

## 4. Discussion

In this project, 37,934 carcasses originating from 318 different agricultural holdings were examined during meat inspection at four different abattoirs. On average, application of the old scheme (n = 17,589) showed that pneumonia was recorded in 50.6% of lungs and pleurisy in 11.6% of the carcasses. With the new scheme (n = 20,345), pneumonia was documented in 44.9% and pleurisy in 11.1% of the animals examined.

The slightly higher rate of pneumonia under the old scheme may reflect that unhealthy batches of pigs were slaughtered and recorded in the extended time period for data collection more frequently compared to those in the new scheme. Analysis of the subset of carcasses evaluated according to both schemes showed no difference in prevalence. Although the introduction of the low-grade level might be understood as more sensitive in contrast to the old approach, the data from the pigs that were assessed in parallel with the both schemes (Figure 3) showed similar frequencies of recorded pathological changes. This confirms that lungs without alterations are distinguished from those with low-grade alterations using both schemes.

Pathological changes in the respiratory tract are a common abnormality observed in this research, which aligns with other studies [11,28,29,30,35]. The prevalence of pneumonia recorded in various studies ranged from 13.3 to 69.1%, and for pleurisy, it ranged from 6.9% to 14.4%. For example, according to a project in Austria in 2016 in which data from 4 million carcasses were analyzed, pneumonia was found in 21.9% of post-mortem examinations, and pleurisy was found in 6.9% [11]. A study from France carried out in 19 slaughterhouses showed that 69.1% of lungs had pneumonia and 14.4% had pleurisy [36]. According to the analysis of Vecerek et al. [35] in the Czech Republic, lung lesions were recorded in 41% of the animals. A study from Italy in 2007 showed that in 3603 pigs divided into 41 batches, 59.6% had pneumonia lesions [37]. By contrast, research from Croatia showed that 13.3% of the pigs had pneumonia and 7.5% had pleurisy [38]. Finally, Čobanović et al. [39] reported from Serbia that 38.3% of the examined barrows and gilts had pneumonia and 6.7% had pleurisy. As the recording of pneumonia and pleurisy is not harmonized between countries, no conclusions on the animal health situation in the different European countries should be drawn.

The results of our study confirmed the expected benefit from the new scheme, showing significant differences in the severity grade of alterations observed on the animal and batch level. Aggregation to the batch level supports interpretation of the health status of the animal group and can guide action by the supervising veterinarian. Among the animals with pneumonia, low-grade forms were observed in 28.4%, followed by the moderate grade in 11.3%, and the rarest was high-grade pneumonia at 5.2%. These results are similar to the study by Mues et al. [40] from Germany, which also recorded the lung changes in a multi-stage system with comparable categories. It showed that 54, 32, 12, and 3% of the lungs had no visible changes and low-, moderate-, and high-grade changes, respectively.

Investigation of correlation between pneumonia and pleurisy showed a weak but significant positive correlation between pneumonia and pleurisy also with regard to severity (Table 1). This is in line with the fact that several agents for respiratory tract infection involve the lung and the pleura during disease progress, but also with the fact that in some cases chronic pleuritis dominates in a later stage of the disease. Whereas *M. hyopneumoniae* and PCV-2 (Porcine circovirus type 2) are some of the most common pathogens involved in lung lesions [40,41,42,43,44], pleural lesions are frequently associated with other etiologic agents, with *A. pleuropneumoniae* being the most frequent [16,17]. In a recent study, Petri et al., 2023 [45] found a strong correlation between the severity scores of pleurisy lesions, with the presence of *P. multocida* and *A. pleuropneumoniae.* For the lung samples, the severity of the lesions was correlated with the presence of *P. multocida* and *M. hyopneumoniae*. Similarly, De Conti et al. [46] found a prevalence of 43.3% of *P. multocida* type A (65/150) in lungs without the occurrence of pleuritis in commercial farms in Brazil, and link to *M. hyopneumoniae* and *P. multocida* type A infections was postulated.

It is noticeable that no pleurisy was recorded in 38% of cases with high-grade pneumonia (Table 1), which does not necessarily lead to condemnation of the carcass and confirms the value of recording both findings separately. The reason for this is presumably because the infections were still in the acute phase, as fibrotic adhesions between the lungs and the pleura typically form only in the chronic stage [47]. The healing, repair, and recovery period of pneumonia can last 2–25 weeks depending on the pathogen, and the phase is prolonged by secondary bacterial infections. By contrast, the hematogenous dissemination of bacteria mainly causes pleuritis in pigs and leads to the fibrous adhesions, which are commonly observed in swine at abattoirs [48].

Differences in prevalence rates might indicate that the recording was not carried out in a comparable way by the individual meat inspectors. This might be true in particular for the data generated by the old scheme, as applying the new scheme was preceded by training sessions in which agreement on the documented severity of each alteration was discussed and the homogeneity of recording the findings of the various veterinarians working in different slaughterhouses was checked for consistency. Furthermore, a simple but standardized reporting form was used by all meat inspectors. Previous studies showed a strong variation in the recording of pathological changes among the observers. For example, the work of Eckhardt et al. [49] showed a significant difference in the accuracy of assessment of multi-stage pneumonia cases. These data were collected within a 22-month period and involved 13 official veterinarians. Another study from Austria [50] showed that there was typically a larger variation in assessment among the official veterinarians, especially when there were several grades to choose from for one of the pathological changes. Similar results were obtained in a study from Germany [27], which found a concordance of only 25% for lung lesions and 38% for pleurisy using the Kendall concordance coefficient. In Denmark, the study by Bonde et al. [51] showed that regular meat inspection in general lacked sensitivity compared to the researcher evaluation.

One reason for the wide variation in results among official veterinarians might be the incorrect differentiation between slaughter-related artifacts and actual pneumonic changes, which was worked through in detail in the study by Nathues et al. [52]. The study by Steinmann et al. [25] also showed a large deviation of more than 75% between the examiners in the detection of moderate pneumonia. The cause of the disagreement was the misinterpretation of technical artifacts that were incorrectly assessed as lung lesions by meat inspectors. However, a post-training showed a significantly improved reliability of lung lesion evaluation. In particular, the disagreement in classifying moderate cases was decreased to a total deviation of 15% from the reference.

The main reason for the differences observed with regard to the batch- or slaughterhouse-specific prevalence rates was confirmed to be the differences in the health status of the animals delivered for slaughter. Unfortunately, no additional data on the farms of origin were available to explain better the variability observed. To approach this further, batch-specific analyses showed that there are significant differences between the slaughter batches delivered from one farm to one slaughterhouse and evaluated by one trained meat inspector. Statistical analysis confirmed that farm of origin was the factor with the highest impact on the recorded prevalence of pneumonia or pleurisy, followed by the batch, an observation which was also shown in previous studies [11]. To display the observable variability between slaughter batches, farms delivering high numbers of slaughter batches, each with a reasonable number of animals, were plotted separately on the basis of data collected with the old or new scheme (Figure 6 and Figure 7). These data showed that groups of animals may display some heterogeneity with regard to the frequency of pneumonia and pleurisy from week to week, even when fattened on the same farm with the same management and slaughtered at the same abattoir.

The high rate of findings across various agricultural holdings indicates a strong potential for improving management practice. Gray et al. 2021 [53] stated that it is important that links between farm characteristics, animal performance, and animal health be recognized and understood for sustainable livestock production. In their study, they showed that meat inspection data are more valuable at a finer resolution and could be a useful tool in monitoring batch-level pig health in the future. In a study performed by Kuberka et al. 2024, litter bedding in weaners was associated with a lower EP-like lesion rating than slatted floors, which have a significantly higher score. Furthermore, the transition from a single- to a two-phase fattening system also led to significantly fewer EP-like lesions [54] emphasizing the need for a detailed investigation of the management system in the next step. Vaccination could offer an effective approach to handle respiratory disease problems. Studies have shown that pigs that are vaccinated have fewer and less severe lung lesions, and the effectiveness of transmission of the pathogen is also greatly reduced [55,56], showing further examples for future investigation where data from meat inspection can be helpful to assess the benefit from such programs.

The benefits of improved supervision of animals are reduced animal welfare issues and economic losses as diseases in the respiratory tract slow down growth, reduce fattening performance, and increase mortality in the herd [44,57]. One study showed that the presence of EP-like lesions reduced carcass weight by 1.26 kg and, in the case of pleurisy, by 1.25 kg [58]. Kuchling et al. (2015) also showed an average daily weight gain decrease of 13 g in the case of severe pneumonia and 7 g in the case of visceral pleurisy. This decline increased with the frequency of recorded post-mortem findings [21].

In the course of this project, it became apparent that the implementation of the new scheme can be realized in a resource-saving manner. In contrast, a more detailed recording taking into account the type of alterations was considered not feasible in routine. The technical requirements for realization only need an update to the software, more precisely an insertion of the severity levels for easy recording. A training course with a coherence check of the staff is necessary to ensure that a uniform judgment is made when recording the findings. In addition, although not all abattoirs in Austria were included in the project, the involvement of four different abattoirs located in two different federal states and working with different software systems confirmed the applicability of the proposed approach on a national scale. The practicability of the proposed new scheme combined with the benefit for improved feedback to farmers and veterinarians was acknowledged by the competent authority, which decided to implement the scheme on a routine basis once the technical requirements are met.

Although this study provides valuable data and showed promising results in the implementation of the new system, there are certain limitations. Comparative data for slaughter batches were limited, as consent could not be collected from all farmers. More importantly, no information on the type of farming, the management of the various 318 agricultural holdings, as well as the disease records of the animals was available, which would have allowed for a more detailed analysis and interpretation of the outcomes. Something that is also notable is that the data were collected in a specific time period and not spread over the whole year as in some similar studies. The study also confirmed the benefits of and needs for a well-described standardized system with training and coherence checks, in order to avoid subjective deviations among the assessors when performing evaluations of pathological manifestations.

## 5. Conclusions

This study showed that the new scheme provides improved information for more detailed feedback to farmers and veterinarians and targeted action. It emphasizes the important role of veterinarians during meat inspection in assessing the health of the animals as their responsibilities are currently much broader, including a focus on food hygiene. High-quality meat inspection data can meet the needs identified by experts in organizing better feedback from slaughterhouses: high-quality, reliable, clear information on the health status indicators of the slaughtered animals. These data should be delivered in real time to farmers and their advisors, for example, on lung lesions and pleurisy [59]. To pool strengths and leverage costs, this approach should be combined with an enhanced integration of animal health data in a collaborative approach with industry and academia where possible [60,61]. Such a method may also provide critical information in near real time using data standards and verification protocols to support early warning for health threats, early detection of health events, and overall situational awareness of disease activity [59].

Valid meat inspection data may be also used to address growing consumer concerns on issues of animal health and welfare, as well as food safety, as they can reflect whether food-producing animals are being raised in an ethical and healthy manner. For this purpose, valid animal health and food safety data at the slaughterhouse level might be transparently made available to the public [60].

Combined with the targeted training of meat inspectors and applied on a regular basis in Austria in the upcoming period on all 5 million pigs slaughtered annually [62], this method will provide a valuable tool for generating guidance for on-farm interventions to help reduce the prevalence of respiratory diseases. It supports improving animal husbandry and animal welfare and contributes to sustainable improved animal farming.

## Figures and Tables

**Table 1:** Cross-tabulation of the frequency of the various pneumonia/pleurisy combinations.

	No Pneumonia	Low-Grade Pneumonia	Moderate-Grade Pneumonia	High-Grade Pneumonia
No pleurisy	57.2%	38.6%	29%	38%
Low-grade pleurisy	28.8%	42.1%	16.8%	8%
Moderate-grade pleurisy	10.5%	14.7%	36.2%	10.3%
High-grade pleurisy	3.5%	4.6%	18%	43.7%

**Table 2:** Percentage of variance explained by the individual factors.

Factor	Pneumonia	Pleurisy
Batch	4.14	2.29
Farm	14.27	18.54
Meat inspector	0.09	0.09
Slaughterhouse	−6.62 ×10^−6^	−1.73 × 10^−3^

## Data Availability

Data available on request from the authors due to privacy and legal restrictions.
